# Supplementary material for: Content-rich biological network constructed by mining PubMed abstracts
Source: BMC Bioinformatics. 2004 Oct 8;5:147. doi: 10.1186/1471-2105-5-147 (PMC528731; doi:10.1186/1471-2105-5-147)
Supplement: Additional File 2 — The original results of the above study (non-essential files are deleted to keep the file size under the limit set by BMC bioinformatics). [file 1471-2105-5-147-S2.bz2 › chilibotAdditionalFile2/dip05/766ID9732274E18155/html/ENO_PNP.html]

 


 **ENO** and **PNP** 
  
Found 93 abstracts in PubMed, retrieved 05.  
 

 What does Google say? 
 PDF only 
| .edu only 

---

**Interactive relationship** (e.g. stimulation, inhibition, etc)

**Neutral relationship**- RNase E is a large multidomain protein with N terminal ribonucleolytic activity, an RNA binding domain and a C terminal scaffold that binds PNPase  [ **PNP** ] , enolase  [ **ENO** ]  and RhlB.  Ref: 12035760 Biochem Soc Trans, 2002

**Non-interactive relationship** (e.g. studied together, co-existance, homology, etc.)

- However, binding of RhlB or PNPase  [ **PNP** ]  to enolase  [ **ENO** ]  was not detected under the same conditions.  Ref: 12181321 J Biol Chem, 2002
- Using an E. coli two hybrid system, together with BIAcore apparatus, we investigated the ability of three proteins, polynucleotide phosphorylase  [ **PNP** ]  PNPase  [ **PNP** ] , RhlB RNA helicase, and enolase  [ **ENO** ] , a glycolytic protein, to interact physically and functionally independently of RNase E.  Ref: 12181321 J Biol Chem, 2002
